# Repeatability of quantitative MR fingerprinting for T_1_ and T_2_ measurements of metastatic bone in prostate cancer patients

**DOI:** 10.1007/s00330-024-11162-z

**Published:** 2024-11-06

**Authors:** Mihaela Rata, Matthew R. Orton, Nina Tunariu, Andra Curcean, Julie Hughes, Erica Scurr, Matthew Blackledge, James d’Arcy, Yun Jiang, Vikas Gulani, Dow-Mu Koh

**Affiliations:** 1 Department of Radiology, MRI Unit, The Royal Marsden NHS Foundation Trust, London, UK; 2 Division of Radiotherapy and Imaging, The Institute of Cancer Research, London, UK; 3 Department of Radiology, University of Michigan, Ann Arbor, Michigan USA

**Keywords:** Bone, Prostate cancer, Metastasis, MR fingerprinting

## Abstract

**Objectives:**

MR fingerprinting (MRF) has the potential to quantify treatment response. This study evaluated the repeatability of MRF-derived T_1_ and T_2_ relaxation times in bone metastasis, bone, and muscle in patients with metastatic prostate cancer.

**Materials and methods:**

This prospective single-centre study included same-day repeated MRF acquisitions from 20 patients (August 2019–October 2020). Phantom and human data were acquired on a 1.5-T MR scanner using a research MRF sequence outputting T_1_ and T_2_ maps. Regions of interest (ROIs) across three tissue types (bone metastasis, bone, muscle) were drawn on two separate acquisitions. Repeatability of T_1_ and T_2_ was assessed using Bland-Altman plots, together with repeatability (r) and intraclass correlation (ICC) coefficients. Mean T_1_ and T_2_ were reported per tissue type.

**Results:**

Twenty patients with metastatic prostate cancer (mean age, 70 years ± 8 (standard deviation)) were evaluated and bone metastasis (*n* = 44), normal-appearing bone (*n* = 14), and muscle (*n* = 20) ROIs were delineated. Relative repeatability of T_1_ measurements was 6.9% (bone metastasis), 32.6% (bone), 5.8% (muscle) and 21.8%, 32.2%, 16.1% for T_2_ measurements. The ICC of T_1_ was 0.97 (bone metastasis), 0.94 (bone), 0.96 (muscle); ICC of T_2_ was 0.94 (bone metastasis), 0.94 (bone), 0.91 (muscle). T_1_ values in bone metastasis were higher than in bone (*p* < 0.001). T_2_ values showed no difference between bone metastasis and bone (*p* = 0.5), but could separate active versus treated metastasis (*p* < 0.001).

**Conclusion:**

MRF allows repeatable T_1_ and T_2_ measurements in bone metastasis, bone, and muscle in patients with primary prostate cancer. Such measurements may help quantify the treatment response of bone metastasis.

**Key Points:**

***Question*** *MR fingerprinting has the potential to characterise bone metastasis and its response to treatment.*

***Findings*** *Repeatability of MRF-based* *T*_1_ *measurements in bone metastasis and muscle was better than for* *T*_2_.

***Clinical relevance*** *MR fingerprinting allows repeatable* *T*_1_ *and* *T*_2_ *quantitative measurements in bone metastasis, bone, and muscle in patients with primary prostate cancer, which makes it potentially applicable for disease characterisation and assessment of treatment response.*

## Introduction

Magnetic resonance fingerprinting (MRF) is a rapid imaging technique that yields multiple inherently co-registered quantitative images from a single acquisition [1]. MRF applications in oncology (covering diagnosis, characterisation and response monitoring) [2] have been recently assessed in the brain [3], liver [4], ovary [5], prostate [6, 7], breast [8], kidney [9], or bone metastases [10, 11]. Few studies have reported the repeatability of MRF in brain [12, 13], breast [14], or prostate [15, 16]; note that all these relevant studies evaluated normal tissues only.  The MRF repeatability of normal bone or bone metastasis has not been previously established.

Bone is the most common metastatic site in patients with prostate cancer and accounts for significant mortality and morbidity. The accurate evaluation of the response of bone disease to treatment remains challenging despite the use of Response Evaluation Criteria in Solid Tumours (RECIST 1.1) [17], MD Anderson criteria [18] or the prostate-cancer working group criteria [19, 20]. This is because the inherent limitations of these criteria in detecting early treatment effects, as well as the relative insensitivity of computed tomography (CT), Technetium-99m bone scintigraphy, and morphological MRI for identifying the response of bone metastases to treatment [21].

Whole-body MRI (WB-MRI), using diffusion-weighted and Dixon imaging, has emerged as a potential response biomarker in metastatic bone disease [22]. The Apparent Diffusion Coefficient (ADC) of bone disease has been shown to significantly increase in responders to effective therapy [23]. However, the temporal and magnitude of ADC increase in responders remain uncertain, so other complementary MRI measurements that can inform on the tumour biology, such as T_1_ and T_2_ relaxivities of tissue, can complement the assessment. Viable tumours often have long T_1_ and T_2_ values and significant reduction of these values can be indicators of treatment response in some chemotherapies [24–26].

Standard quantitative MR techniques for measuring T_1_ (such as inversion-recovery sequences with multiple TIs) and T_2_ (spin echo sequences with multiple TEs) are not used routinely in clinical practice due to their lengthy acquisition times (e.g. recommended imaging protocols in [27]) and lack of co-registration. The arrival of fast MRF techniques allows such measurements in bone disease [10, 11]. Whilst absolute accuracy remains the gold-standard performance criterion, measurement repeatability is also important when assessing the utility of such quantitative measures in clinical practice. This study aimed to evaluate the repeatability of MRF-derived T_1_ and T_2_ relaxation times in bone metastasis in patients with prostate cancer. In addition, repeatability was also assessed in normal-appearing bone and muscle in order to provide complementary information for this technical evaluation, as these may have a bearing on disease characterisation.

## Materials and methods

This study included data from patients and a test object.

### Patients

**Fig. 1:**
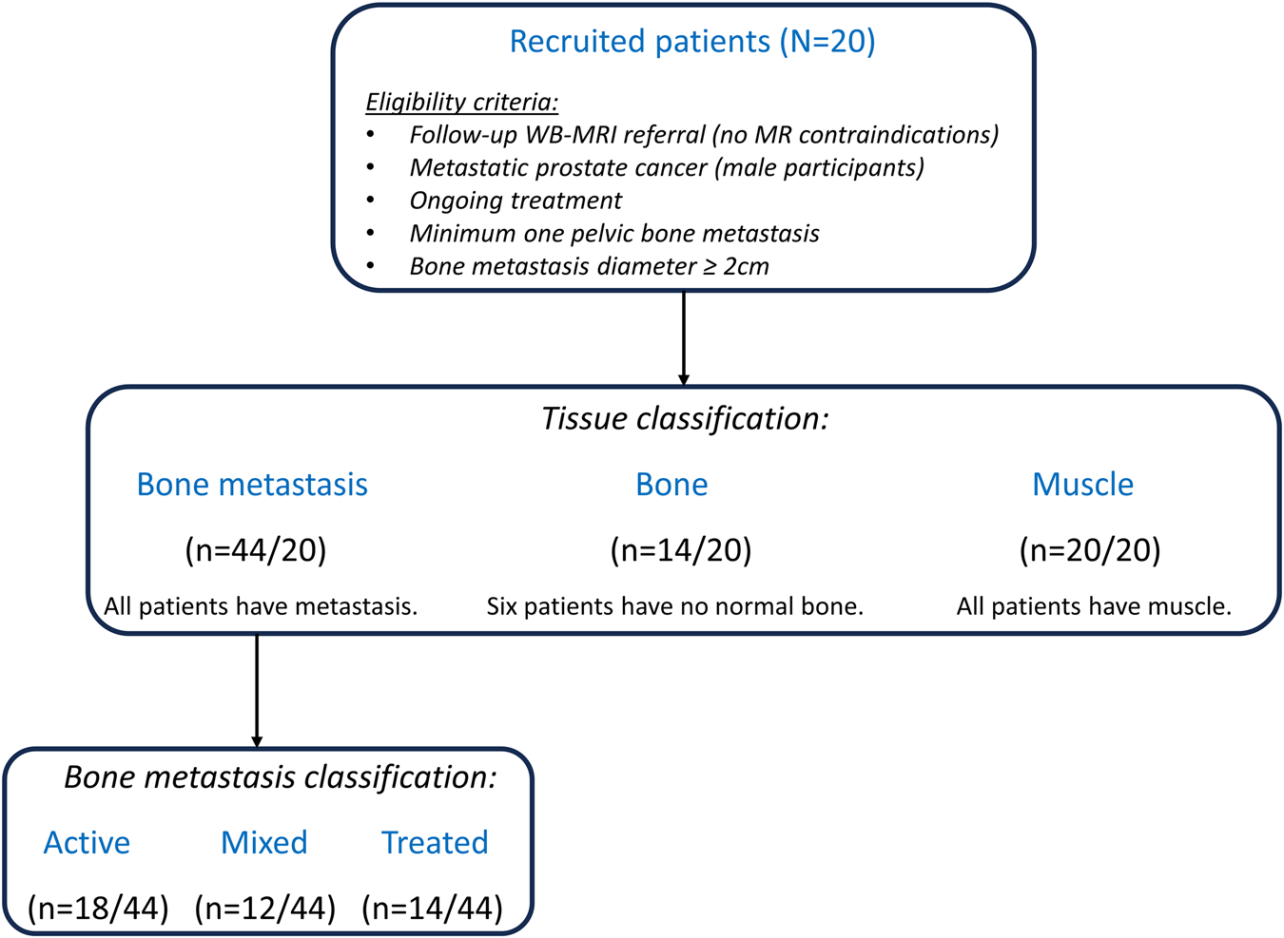
Flow chart of the patient cohort (including eligibility criteria) and classification of various other sub-cohorts. *N* = patient cohort size; *n* = sub-cohort size

Between August 2019 and October 2020, 20 consecutive patients were imaged under a prospective study approved by our institutional clinical review board and national ethics committee. Written informed consent was waived as per study protocol which allowed for the evaluation of a new MRI sequence for up to 15 min based on verbal consent alone. Demographics of the patient cohort and region of interest (ROI) information are presented in Table 1. Figure 1 presents the recruitment flow chart and the sub-cohort splitting. The inclusion criteria were: (1) male patients diagnosed with metastatic prostate cancer; (2) scheduled for a prospective WB-MRI study (at any time point of their cancer treatment); (3) previous MRI showed at least one known bone metastasis within the pelvis (diameter ≥ 2 cm). The exclusion criterion was: (1) patients with known implants in the pelvis, e.g. hip replacement. Two patients were later excluded from the analysis and two others were further recruited to complete the 20-patient cohort. The exclusions were: (1) the pelvic metastasis on current MRI was smaller than the 2-cm diameter threshold, and (2) the positioning of the repeated MRF volume did not slice match the original MRF volume due to technical errors.

**Table 1:** Demographics of the 20-patient study cohort

Participants	Age (years)	ROI			Bone metastasis ROI details		
		Bone metastasis	Bone	Muscle	Count	Type	Slices
1	63	√	√	√	2	1 mixed, 1 treated	4
2	68	√	√	√	2	1 mixed, 1 treated	4
3	69	√	x	√	5	4 active, 1 mixed	3
4	65	√	x	√	2	2 treated	3
5	83	√	√	√	3	3 treated	5
6	58	√	x	√	3	2 active, 1 mixed	3
7	72	√	√	√	2	2 treated	4
8	80	√	x	√	3	3 mixed	4
9	67	√	√	√	1	1 treated	4
10	61	√	√	√	2	1 active, 1 mixed	2
11	80	√	x	√	2	1 mixed, 1 treated	5
12	60	√	√	√	1	1 active	2
13	77	√	√	√	6	2 mixed, 3 treated, 1 active	4
14	78	√	√	√	1	1 mixed	2
15	80	√	√	√	3	3 active	3
16	81	√	x	√	1	1 active	5
17	59	√	√	√	1	1 active	1
18	77	√	√	√	1	1 active	5
19	65	√	√	√	1	1 active	4
20	65	√	√	√	2	2 active	4
Cohort summary (*N* = 20)	Mean ± standard deviation	Count/total	Count/total	Count/total	Total	Count for metastasis type	Total
	70 ± 8	20/20	14/20	20/20	44	18 active; 12 mixed; 14 treated	71

All patients had metastatic prostate cancer with extensive pelvic bone disease and were consecutively enrolled in this study. Information about the delineation of ROIs within normal tissue (bone and muscle) and metastatic bone (active, mixed, treated disease) is given

One bone ROI was drawn on one slice only covering the pelvis area

One muscle ROI was drawn on one slice only covering the pelvis area

Bone metastasis ROIs were drawn on up to five slices covering the pelvis area

*ROI* region of interest

A prototype MRF sequence (see Table 2) was run twice per patient during the same-day scan session. The first run continued immediately after the clinical protocol. Afterwards, the patient was removed from the table and allowed to briefly walk/stretch within the scanner room, before returning to the table to be repositioned for a second MRF scan. Care was taken to position the patient and the pelvic body-array coil as closely as possible to the first scan.

**Table 2:** MR parameters of the assessed MR fingerprinting sequence. Two additional functional sequences (diffusion-weighted imaging and T_1_ Dixon imaging) used to aid delineation and radiological classification of disease ROI (active, mixed, treated) are also detailed

MRI parameters	Sequences		
	MRF	DWI	Dixon
	2D gradient echo (fisp)	2D single-shot echo planar imaging	3D gradient echo (flash)
Acquisition plane	Axial	Axial	Axial
Breathing mode	Free breathing	Free breathing	Free breathing
Total acquisition time (min:s)	03:03	03:35	00:16
Parallel acquisition	-	Grappa (2 × 32)	Caipirinha (2 × 2)
Number of averages	1	Per b value: 2, 2, 4	1
b values (s/mm^2^)	-	50, 600, 900	-
Reconstructed voxel size (mm^3^)	1 × 1 × 5	1.6 × 1.6 × 5	0.8 × 0.8 × 5
Slice thickness (mm)	5	5	5
TR (ms)	11.2	6150	7.63
TE (ms)	2.21	64	2.39, 4.77
TI (ms)	21	180	-
Flip angle (˚)	0–50	90	19
Slices	5	40	40
recon. matrix (FE x PE)	400 × 400	268 × 216	512 × 384
FOV (mm^2^)	400 × 400	430 × 346	430 × 322
k-space trajectory	Spiral	Cartesian	Cartesian
Receiver bandwidth (Hz/Pixel)	390	2330	400
Fat suppression	None	STIR (short tau inversion recovery)	Dixon method

*TR* repetition time, *TE* echo time, *TI* time of inversion, *FE* frequency encoding, *PE* phase encoding, *FOV* field of view

### Test object

**Fig. 2:**
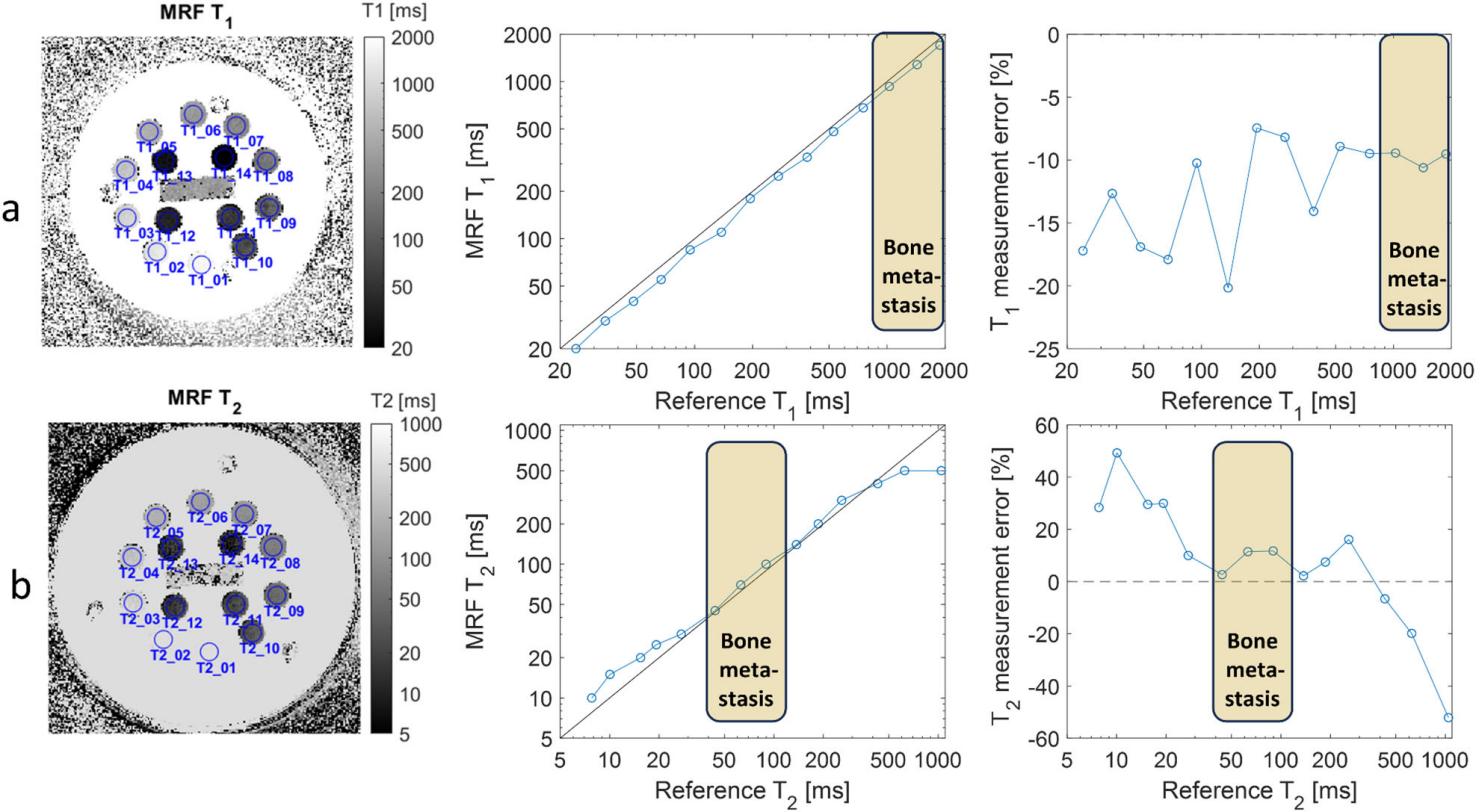
MRF measurements of T_1_ (**a**) and T_2_ values (**b**) from a commercially available test object covering a large range of values (two 14-vial arrays for T_1_ and T_2_) at 20 °C room temperature. The reference values were taken from the test object documentation. Direct comparison and percentage error plots are presented. The cream rectangle indicates the range of values relevant to bone metastasis

The prototype MRF sequence was run on a commercially available test object (NIST/ISMRM phantom, System Standard Model 130) covering a wide range of T_1_ and T_2_ values, see Fig. 2. The test object [27] is a 20-cm spherical phantom that includes three 14-vial arrays specifically designed to assess T_1_ (water doped with NiCl_2_), T_2_ (water doped with MnCl_2_), and proton density M_0_ (water doped with D_2_O). At 1.5 T and 20 °C room temperature, the range of references varies across vials from 24 to 1879 ms for T_1_, and from 8 to 1044 ms for T_2_. These 14 calibrated reference values for T_1_ and T_2_ were supplied within the test-object documentation.

### MR protocol

All imaging was performed on a 1.5-T MAGNETOM Aera (Siemens Healthcare), using a 3-min prototype 2D MRF-FISP sequence [3] with Gadgetron reconstruction [16], which yields maps of T_1_ and T_2_ relaxation times, and the proton density M_0_. Five contiguous axial 5mm-thick slices were positioned over the pelvis to include lesions that appeared hyper-intense on high b-value images and hypo-intense on the fat-only Dixon images available from the WB-MRI protocol. Detailed MR protocol parameters for the MRF, diffusion-weighted imaging (DWI), and T_1_-weighted Dixon are presented in Table 2.

### Data analysis

**Fig. 3:**
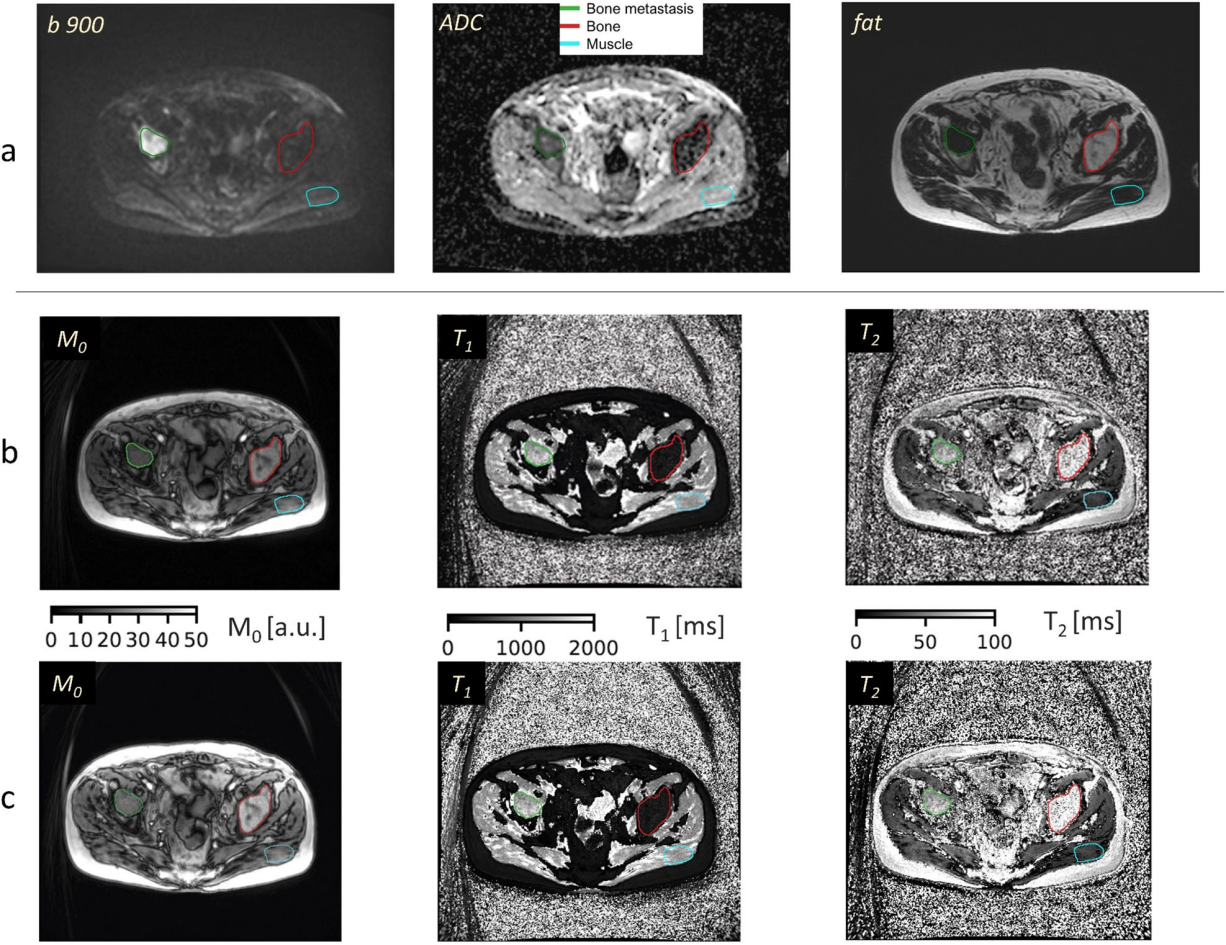
**a** Matched axial parametric maps derived from DWI and Dixon acquisitions for a 65-year-old patient, helping to differentiate between normal and metastatic bone. **b**, **c** Example of two repeated MRF measurements of M_0_, T_1_ and T_2_ derived from the same patient demonstrating an active bone metastasis in the right iliac bone (green), normal-appearing bone (red) and gluteus muscle (blue). *ADC*, apparent diffusion coefficient

For the patient analysis, ROIs were drawn for three regions per subject (bone metastasis, normal-appearing bone, and gluteus muscle) on the MRF M_0_ image, using the DWI and fat-only Dixon images as a visual guide, see Fig. 3. ROIs drawn on the first MRF scan were copied to the second scan and, if needed, manually adjusted to accommodate any minor changes in pose and position of patient. The same window level (WL) and window width (WW) were used for delineating the two sets of MRF M_0_ images per patient; these values were typically WL 50 and WW 100. Bone metastasis ROIs, with a diameter ≥ 2 cm, were drawn on multiple slices (up to the available maximum of five slices) to ensure maximal tumour volume coverage. Where possible, on a single slice, a normal-appearing bone ROI was drawn on the contralateral side relative to the bone metastasis. Both ROIs for normal tissues (bone and muscle) were drawn on the same single slice; that specific slice included an ROI for bone metastasis as well (Fig. 3). In cases where more metastases were included per individual, each metastasis was considered separately. For the purpose of subsequent analyses, a metastasis was considered active if it showed ADC value < 1000 mm^2^/s, treated if ADC value > 1500 mm^2^/s and mixed if it contained elements of both. All ROIs were delineated by an experienced physicist (M.O. or M.R., each > 10 years experience) working under the supervision of a senior radiologist (N.T., > 15 years experience). All delineation was performed on anonymised data that were archived on a project-specific XNAT database [28]. ROIs were drawn using Horos software (Horosproject.org), saved as DICOM-RT structure sets using a pyOsiriX plugin [29], and image statistics were obtained using Python v3.6.8. Mean values for T_1_ and T_2_ measurements were computed from all voxel values for each type of tissue, for each patient and for each separate scan. T_1_ and T_2_ cohort mean values were reported for each type of tissue and each scan.

For the test-object analysis, circular regions of interest (ROIs) were delineated on each of the 14 vials to derive MRF-based T_1_ and T_2_ values, see Fig. 2. These measured values were compared with tabulated references available from the test-object documentation. Relative measurement errors were reported.

### Statistics

Repeatability was visually assessed with Bland-Altman plots [30], and statistically assessed using the repeatability (r), and intraclass correlation (ICC) coefficients. Several cohort statistics parameters were reported across three regions (i.e., metastasis, normal bone, muscle) and three types of metastases (treated, mixed and active): mean across the two repeated measurements, standard deviation, mean bias, between subjects standard deviation (SD_B_), within subjects standard deviation (SD_W_) and limits of agreement (LoA). Derived parameters such as r, r% and ICC were calculated as: r = 1.96 × 1.41 × SD_W_; r% = 100 × (r/mean); ICC = SD_B_^2^/(SD_B_^2^ + SD_W_^2^); LoA = bias ± r. The ICC values were interpreted according to [31] as: poor (< 0.5), moderate (0.5–0.75), good (0.75–0.9) and excellent (> 0.9). T_1_ and T_2_ values were compared between bone metastasis and bone using *t*-test and between the three types of metastases using Anova test (followed by post-hoc analysis with Bonferroni correction). A *p*-value < 0.05 was considered significant for all tests excluding the post-hoc analysis (that used a 0.05/3 = 0.017 threshold for Bonferroni-corrected data).

## Results

### Patients

The study cohort included 20 consecutive male patients (mean age of 70 years) who were previously diagnosed with prostate cancer and had a measurable bone metastasis within the pelvis, see Table 1.

The MRF acquisition allowed quantitative T_1_ and T_2_ measurements in 3 min. A total of 44 bone metastases, 14 normal-appearing bone, and 20 muscle ROIs were delineated across the cohort. Six individuals had no normal-appearing bone marrow within the 3D volume covered by the MRF acquisition (25 mm thick). The 44 bone metastases (range 1–6 lesions per patient) were categorised by the radiologist (N.T.) into three classes based on DWI and Dixon characteristics: active/hypercellular (*n* = 18), heterogenous/mixed with areas of hypercellular and low cellularity (*n* = 12) and treated/low cellularity disease (*n* = 14). The cohort means of the ROI volumes per tissue type were: 14.03, 2.49, 2.80 mL for metastasis, normal-appearing bone, and muscle, respectively. As exemplified in Fig. 3b, c, a good agreement between positions of the two MRF acquisitions was obtained when delineating ROIs. Bone metastasis (green overlay) was radiologically confirmed by high signal on high-b-value image, low ADC and low-fat content on fat image. On MRF-derived maps, the bone metastasis showed intermediate signal on both T_1_ and T_2_, whilst normal bone (red overlay) had low T_1_ and very high T_2_ values compatible with fatty tissue.

**Fig. 4:**
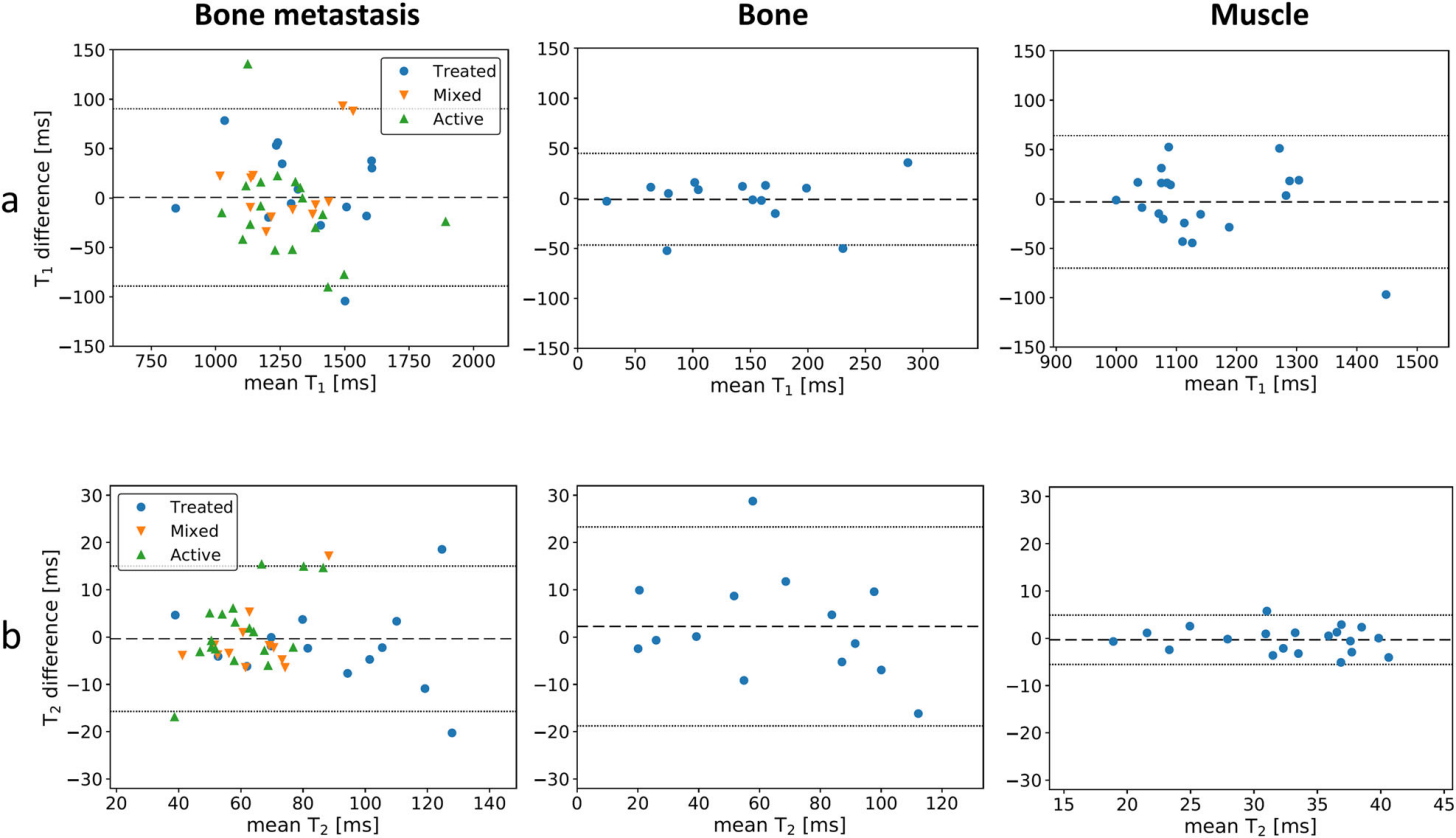
Bland-Altman plots of MRF-derived T_1_ (**a**) and T_2_ measurements (**b**) across the 20-patient cohort, for each tissue type (bone metastasis, normal-appearing bone, muscle). The 44 bone metastases derived from the whole cohort also have different symbols to identify the radiological classification of disease: active, mixed, and treated. Mean bias and limits of agreement are plotted as dashed lines. The same vertical scale is displayed for all three tissues for T_1_, and T_2_, respectively

Bland-Altman plots are shown in Fig. 4 for all three tissue types. Relevant repeatability statistics are listed in Table 3 across the three types of tissues (metastasis, normal-appearing bone, and muscle) and also across radiologically classified metastases (treated, mixed and active). The T_1_ and T_2_ biases of the repeated measurements across all twelve measurements were not statistically significant (*t*-test; range of *p*-values was 0.30 to 0.93). The largest r% values were found for the normal-appearing bone tissue for both relaxation parameters (32.2–32.6%), whilst muscle demonstrated the smallest r% values (5.8% for T_1_ and 16.1% for T_2_). Similar to muscle, bone metastasis had a much smaller r% for T_1_ measurements (6.9%) than that for T_2_ (21.8%). Any of the three classes of bone metastases demonstrated similar behaviour, with T_1_ (r% range: 5.9–7.2%) being more repeatable than T_2_ (r% range: 18.7–25.5%). Across all twelve measurements, ICC for T_1_ measurements was excellent (> 0.94); ICC of T_2_ measurement was good (> 0.82) or excellent.

**Table 3:** Summary results for the MRF-derived T_1_ and T_2_ values and their repeatability statistics for bone metastasis (including treated/mixed/active metastasis), normal bone and muscle tissues

Region	Measured parameter	Mean	SD	Bias	SD_B_	SD_W_	r	r%	ICC	Lower LoA	Upper LoA
		ms	ms	ms	ms	ms	ms	%	a.u.		
Metastasis	T1	1300.6	195.1	0.6	192.9	32.4	89.4	6.9	0.97	−88.8	90.1
	T2	70.2	22.2	−0.3	22.0	5.5	15.3	21.8	0.94	−15.7	15.0
Treated metastasis	T1	1331.4	222.8	7.4	214.7	31.4	86.7	6.5	0.98	−79.2	94.1
	T2	88.4	27.8	−2.2	26.8	6.0	16.6	18.7	0.95	−18.7	14.4
Mixed metastasis	T1	1280.7	164.0	12.2	157.1	27.5	76.1	5.9	0.97	−63.8	88.3
	T2	63.5	12.6	−0.9	12.0	4.4	12.2	19.3	0.88	−13.1	11.4
Active metastasis	T1	1290.0	199.3	−12.4	193.7	33.8	93.4	7.2	0.97	−105.8	80.9
	T2	60.5	12.4	1.4	12.1	5.6	15.4	25.5	0.82	−14.0	16.8
Bone	T1	139.8	70.6	−1.0	68.0	16.5	45.6	32.6	0.94	−46.6	44.7
	T2	65.1	31.1	2.2	30.0	7.6	21.0	32.2	0.94	−18.7	23.2
Muscle	T1	1145.5	114.8	−3.0	111.9	24.2	67.0	5.8	0.96	−70.0	63.9
	T2	32.5	6.3	−0.3	6.1	1.9	5.2	16.1	0.91	−5.5	4.9

Bias = mean2 − mean1

*SD* standard deviation, *SD*_*B*_ standard deviation between subjects, *SD*_*W*_ standard deviation within subjects, *SD* standard deviation, *r* reproducibility coefficient, *ICC* intraclass correlation coefficient, *LoA* limit of agreement

**Fig. 5:**
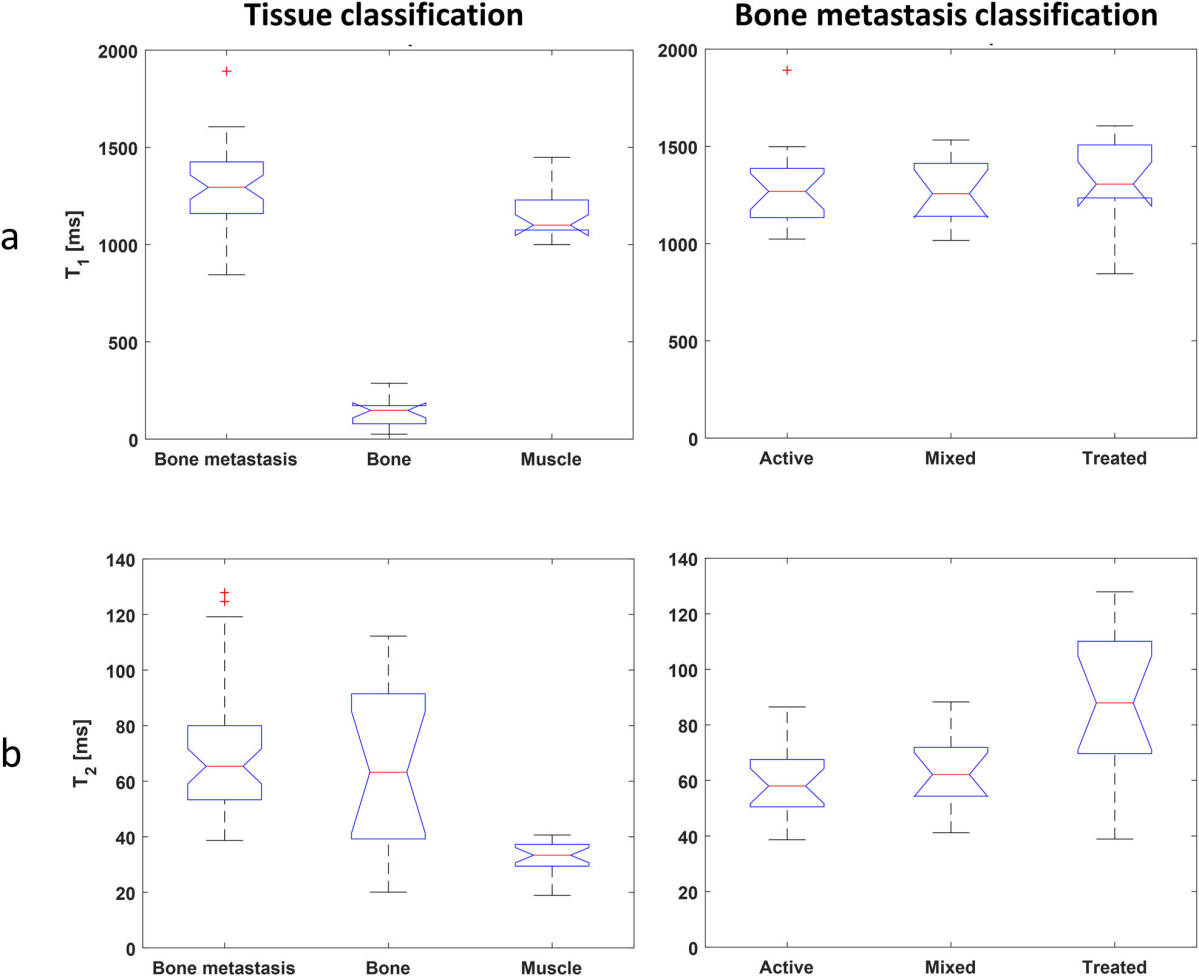
Boxplots of MRF-derived T_1_ (**a**) and T_2_ measurements (**b**) across the 20-patient cohort, for each tissue type (first column) and the bone metastasis sub-cohorts (second column). Boxplot legend: red central line = median; blue line limits = 25% and 75% percentiles; black line limits = most extreme data points that are not outliers; red cross = outlier. Notches are introduced to ease visual comparison between groups: non-overlapping notches suggest statistically significant different medians between groups. Inverted notches visible for three T_1_ sub-cohorts suggest a larger confidence interval for the median value (than the quartile) in these cases. The same vertical scale is displayed for all tissues for T_1_, and T_2_, respectively

Boxplots of measured T_1_ and T_2_ across tissue and bone metastases are shown in Fig. 5, and *p*-values of statistical tests are presented in Table 4. T_1_ values were very well separated between bone metastasis and bone (1301 vs. 140 ms, *t*-test, *p*-value < 0.001). By contrast, T_2_ values did not allow differentiation of bone metastasis and normal bone (70 vs 65 ms, *t*-test, *p*-value = 0.50). Across the metastasis values, no clear separation between T_1_ values derived from active, mixed, or treated disease classes was found (Anova, *p*-value = 0.78). However, the equivalent Anova test for T_2_ found a significant difference between the three metastasis classes (Anova, *p*-value < 0.001), with the T_2_ of treated lesions having a significantly higher value than T_2_ of active lesions (88.4 vs 60.5 ms, *t*-test, *p*-value < 0.001) or that of the mixed class (88.4 vs 63.5 ms, *t*-test, *p*-value = 0.009).

**Table 4:** Statistics comparing MRF-derived T_1_ and T_2_ values for tissue type (bone metastasis versus normal bone) and bone metastasis sub-cohorts (active versus mixed versus treated)

Comparison	Measured parameter	Statistics test	
		Type	*p*-value
Metastasis vs. bone	T1	*t*-test	< 0.001*
	T2	*t*-test	0.50
Active vs. mixed vs. treated metastasis	T1	Anova	0.78
	T2	Anova	< 0.001*
Active vs. mixed	T2	Post-hoc analysis (*t*-test and Bonferroni correction)	0.53
Active vs. treated	T2		< 0.001*
Mixed vs. treated	T2		0.009*

* Statistical significance (*p*-value < 0.05 or, for Bonferroni-corrected data, *p*-value < 0.017 = 0.05/3)

### Test object

Overall, the test-object results demonstrated a good correspondence between the MRF-measured parameters and the reference values, in particular for the range of values similar to those measured in bone metastasis (see highlighted range in Fig. 2) where the relative error measurement of T_1_ or T_2_ was less than 10%.

## Discussion

This study showed that fast MRF acquisitions yield quantitative T_1_ and T_2_ measurements with good repeatability in bone metastasis, normal-appearing bone, and muscle in prostate cancer patients. The relative repeatabilities of T_1_ were 6.9% (bone metastasis), 32.6% (normal-appearing bone), and 5.8% (muscle), whilst for T_2_ were 21.8%, 32.2% and 16.1%, respectively. r% values for treated, mixed, or active metastases were similar to those reported for the overall metastasis group. In addition, all corresponding ICCs were > 0.82, suggesting good/excellent agreement between the two measurements. The r% values imply that, within the context of a future treatment efficacity study, proportional changes less than these values could be attributed to measurement error, while excess changes would likely be treatment-related. The relative repeatability of T_1_ (6.9%) and T_2_ parameters (21.8%) is better or of a similar order of magnitude to ADC estimates (12.5%) [32], which is widely used for MRI assessment of bone metastasis.

The highest r% values found in normal bone ROIs can be explained by the inherent heterogeneity of bone microstructure (in particular, its honeycomb-like network of trabecular bone), suggesting that this is a particularly challenging tissue for MRF relaxometry. By contrast, the muscle (the most homogenous tissue assessed here) had the lowest r% values for both T_1_ and T_2_ parameters. Moreover, the T_1_ and T_2_ repeatability of both bone metastasis and muscle tissues were similar, with T_1_ being always more repeatable than T_2_. Overall, our finding agrees with previous repeatability studies for various healthy tissues [12–16] that also reported better repeatability for T_1_ than T_2_. The improvement of both T_1_ and T_2_ repeatability in bone metastasis versus normal bone may be partially explained by the fact that prostate bone metastases are predominantly osteoblastic (sclerotic) [33], i.e., generating increased bone density compared to normal bone.

T_1_ measurements allowed clear differentiation between bone metastasis and normal-appearing bone (*p*-value < 0.001) within our study, in agreement with two previous MRF reports [10, 11] Note, however, that our study included prostate patients that were under treatment or have had previous lines of treatment; therefore, even if their MRI assessment did not detect any active disease, one cannot consider their bone as completely normal, as would be the case for a treatment-naïve patient. In addition, the osteoblastic changes seen in prostate cancers are expected to lead to reduced T_1_ values, which may explain the lower T_1_ found in normal-appearing bone in our study. Within the metastases sub-cohort, the T_1_ values could not separate between active/mixed/treated disease (*p*-value = 0.78), although an existing study [24] suggests that early response to therapy manifests as decreased T_1_ values. Compared with the two existing studies, our estimates for bone metastasis T_1_ are within the previously reported range (1301 ms vs 1195 ms [10] and 1675 ms [11]), whilst much lower values were found for bone in our study (140 ms vs 461 ms [10] or 447 ms for fatty bone [11]). Similar pelvic bone metastases were assessed for our study and [10], whilst [11] derived their findings from the vertebrae of patients undergoing MRF for diagnosis purposes, i.e. prior to any treatment. As mentioned before, we evaluated normal-appearing bone in the same cohort of metastatic prostate cancer patients, whilst studies [10] and [11] evaluated normal bone in non-malignant patients. By comparison, our muscle T_1_ measurements are in line (1145 vs 1100 ms) with a previous report [4].

The T_2_ parameter was less useful for bone metastasis versus normal-appearing bone differentiation as measured values overlapped across these two groups (*p*-value = 0.50). Conversely, within the bone metastases sub-cohort, the T_2_ parameter of the treated metastasis was significantly higher than those of mixed or active classes (*p*-values = 0.009, < 0.001) supporting the use of T_2_ as a treatment response biomarker. Similar behaviour of an increase in T_2_ values for treated versus untreated metastasis was reported in [10]. Our T_2_ measurements are similar to previous reports in bone metastases (70 vs. 58 ms), normal-appearing bone (65 vs. 78 ms) [11] and muscle (33 vs. 44 ms) [4]. Note that, the reported T_2_ in bone metastasis (averaged across three subclasses) of 70 ms was biased upwards by the contribution of an increased T_2_ of treated lesions (88.4 ms). Therefore, the active bone metastases value (60.5 ms) should be considered instead for further literature comparison.

A recent study [34] using SyMRI (an alternative technique to MRF and capable of generating T_1_, T_2_ and PD quantitative maps) highlighted the utility of the PD maps as this metric was shown to be able to differentiate bone lesion changes with treatment more sensitively than T_1_ or T_2_. Our study did not assess the PD parameter, so future work exploring the use of PD maps could help develop bone response biomarkers. Moreover, a comparative study of SyMRI and MRF [35] found similar repeatability for both methods and results comparable with standard references.

Unlike a recent paper [4], our MRF acquisitions did not include fat suppression pulses. Given the presence of both fat and water signals in bone metastases, additional MRF studies looking to separate T_1_ and T_2_ based on fat and water signals could be explored to allow further characterisation of such tissues.

To enable clinical adoption of MRF, three elements are required: (1) a prototype MRF sequence compatible with the available MR scanner; (2) a direct connection with a high-specification computer (to store the MRF dictionary and the pattern reconstruction algorithm allowing fast and reliable MRF-reconstructed maps); and (3) a standardised test-object to enable initial calibration of the sequence on the given scanner. For the prototype sequence and reconstruction used in this work, less than 3 minutes were required to automatically send raw MRF acquisitions to the external computer to generate the MRF maps and return them to the scanner console.

This study had several limitations. First, our results were derived from a small cohort of 20 patients recruited at a single institution on a 1.5-T scanner. However, an increased sample (*n* = 44) for bone metastasis assessment was available, due to most patients having multiple sites of disease. Second, the reported T_1_ and T_2_ values across bone metastasis, normal bone and muscle are derived only from patients undergoing treatment, and these T_1_, T_2_ values (including those of normal-appearing tissues) should be interpreted within this non-naïve treatment context. Nevertheless, the measurement repeatability of T_1_ and T_2_ of all bone metastases appears sufficiently robust for clinical evaluation. Third, this study did not assess the inter-observer variability, as the main aim of the study was to test the repeatability of the MRF measurements while keeping other factors, such as the observers, the same. The additional variability that could arise from differences in observer ROI delineation was beyond the scope of this study. Further studies on larger cohorts from multiple centres with multiple operators (performing lesion segmentation) using a standardised MRF sequence would help establish quantitative biomarkers that can differentiate normal from disease tissue or inform on treatment response [36]. For these purposes, recommendations on standardisation and validation strategies of MRF [37] are important and should be further developed.

In conclusion, fast MRF allows repeatable quantitative T_1_ and T_2_ measurements in bone metastasis, normal-appearing bone, and muscle in patients with primary prostate cancer. T_1_ was found to be able to differentiate between bone metastasis and normal-appearing bone, whilst T_2_ can separate active versus treated metastasis. Further longitudinal study is ongoing to assess the magnitude of treatment-related changes in T_1_ and T_2_ that occur in bone metastases with reference to the repeatability limits reported here and compared to standard quantitative MRI. Further validation of MRF measurement is required to develop new robust response biomarkers for malignant bone disease.

## Abbreviations

ADC: Apparent diffusion coefficient
DWI: Diffusion-weighted imaging
ICC: Intraclass correlation coefficient
MRF: MR fingerprinting
r%: Relative repeatability coefficient
r: Repeatability coefficient
ROI: Region of interest
WB-MRI: Whole-body MRI
